# Barriers and Facilitators Associated With Vaccine Acceptance and Uptake Among Pregnant Women in High Income Countries: A Mini-Review

**DOI:** 10.3389/fimmu.2021.626717

**Published:** 2021-04-26

**Authors:** Xiao Qiu, Heather Bailey, Claire Thorne

**Affiliations:** ^1^ UCL Institute for Global Health, University College London, London, United Kingdom; ^2^ UCL Great Ormond Street Institute of Child Health, University College London, London, United Kingdom

**Keywords:** vaccination, pregnancy, acceptance, hesitancy, influenza, pertussis

## Abstract

Vaccination during pregnancy is a safe and effective intervention to protect women from potentially severe consequences of influenza and reduce risk of influenza and pertussis in their infants. However, coverage remains variable. In this mini-review we update findings from a 2015 systematic review to describe results from recent studies in high income countries on the uptake of influenza and pertussis vaccination in pregnancy, reasons for vaccine hesitancy and barriers to increasing uptake, from maternal and healthcare provider (HCP) perspectives. Studies reported highly variable uptake (from 0% to 78%). A main facilitator for uptake among pregnant women was receiving a recommendation from their HCP. However, studies showed that HCP awareness of guidelines did not consistently translate into them recommending vaccines to pregnant women. Safety concerns are a well-established barrier to uptake/coverage of maternal immunization; 7%-52% of unvaccinated women gave safety concerns as a reason but these were also present in vaccinated women. Knowledge/awareness gaps among pregnant women and lack of confidence among HCPs to discuss vaccination were both important barriers. Several studies indicated that midwives were more likely to express safety concerns than other HCPs, and less likely to recommend vaccination to pregnant women. Women who perceived the risk of infection to be low were less likely to accept vaccination in several studies, along with women with prior vaccine refusal. Findings highlight the importance of further research to explore context-specific barriers to vaccination in pregnancy, which may include lack of vaccine confidence among pregnant woman and HCPs, and policy and structural factors.

## Introduction

Vaccination in pregnancy was first implemented in the 1960s with tetanus toxoid immunization, with strategies of maternal immunization to protect pregnant women and their infants against influenza and pertussis more recently introduced. The potentially severe consequences of influenza in pregnancy (1) and the efficacy of maternal influenza immunization in preventing infection in young infants (2, 3), alongside reassuring safety data (4–7) have driven recommendations for its widespread use (8, 9). In the last decade, maternal pertussis vaccination programs have also been implemented in high income countries (HICs) to protect neonates, who have high risk of severe complications, through passively transferred maternal antibodies, with high effectiveness (10).

Concerns around vaccination remain an issue despite robust evidence on the safety and benefits of vaccination. The term ‘vaccine hesitancy’ is used to refer to “*delay in acceptance or refusal of vaccines despite availability of vaccination services. Vaccine hesitancy is complex and context specific varying across time, place and vaccines. It includes factors such as complacency, convenience and confidence*” (11, 12). A systematic review on vaccine acceptance in pregnancy in 2015 found that concern about vaccine safety was the main factor contributing to vaccine hesitancy, with other common barriers being lack of recommendation from health care providers (HCPs) and poor vaccine knowledge (13).

Our aim is to update these findings with recent studies conducted in HICs in order to describe the uptake of influenza and pertussis vaccination in pregnancy, explore reasons for vaccine hesitancy and discuss barriers to increasing uptake, from maternal and HCP perspectives. We have therefore focused our narrative review on papers published from April 2015 to July 2020.

## Uptake of Influenza and Pertussis Vaccination in Pregnancy

Ten studies reported on uptake, mainly based on maternal self-report. For maternal influenza vaccination the highest uptakes of 78% and 76%, were reported among 984 women in a US study and 101 women in a New Zealand study (14, 15), with a Spanish study reporting 62% uptake among 683 women (16). Two large studies from France with 2045 and 1194 women reported uptakes of 36% and 22% respectively (17, 18), with uptake of 45% among 823 women in Belgium (19) and 16.2% among 197 women in Greece (20). The lowest coverage was found in a study of 743 women in Italy at 6.5% (21). The latter study also had a low pertussis vaccine uptake, at 4.8%. Pertussis vaccination uptake in other studies ranged from 74% in a large survey of 1809 pregnant women in Taiwan (22) and 61% in an Australian study of 537 women (23) to 64% in the study in Belgium (19) to 0% in the study from Greece (20).

Studies on vaccine acceptance (combining intention to vaccinate with actual uptake at the time of survey) included a multi-site UK survey of around 300 pregnant women, where 38% and 56% had been vaccinated for influenza and pertussis respectively, with a further 40% and 36% intending to be vaccinated (24). In a similar sized survey in the USA, acceptance rates were 71% and 76% respectively for influenza and pertussis vaccination (25). In another US study, with a convenience sample of 316 pregnant women in the public health system, 82% said they had received the vaccine or intended to get the vaccine that day (26). A study of 113 pregnant women in Ireland found uptake rates of 31% for pertussis and 42.5% for influenza, with 29% of unvaccinated women reporting that they would take up if discussed and offered (27). Studies based on acceptance rates need careful interpretation because they may over-estimate final uptake, as demonstrated by Bettinger and colleagues’ finding that 36% of women who stated intention to have the influenza vaccine had not done so by delivery (28). Self-reported actual uptake may also be over-reported (15). Discrepancies are likely to reflect various factors, including social desirability bias and unforeseen barriers to uptake.

## Influence of HCP Offer and Recommendation

Knowledge of availability of influenza and pertussis vaccination in pregnancy is a pre-requisite for women to decide to vaccinate or not, and for a high proportion of women such knowledge is only gained when they are offered vaccination by an HCP. Consistent with previous findings (13), receiving a HCP recommendation was a main facilitator of vaccine uptake among pregnant women in recent studies, and its absence was the pre-eminent barrier reported among unvaccinated women (18, 20, 21, 25, 28–31). To illustrate, in an Italian survey 62% of vaccinated women said that HCP recommendation was the main facilitator of vaccination, whilst 81% of unvaccinated women reported no HCP recommendation as the main barrier experienced (21), while influenza vaccination uptake was 47% in women who reported being recommended to vaccinate by a HCP versus 3% in those who did not in a French study (17). Two studies (from Australia and the US) found that women receiving an HCP recommendation for pertussis vaccination had 10-fold greater odds of being vaccinated compared with those who did not (23, 25).

The importance of HCPs’ recommendation has led several recent studies to specifically investigate recommendation behaviors among HCPs, and vaccine knowledge and attitudes that may underpin these. **Table 1** shows the proportion of HCPs reporting that they recommend influenza and/or pertussis vaccinations to pregnant women or informed their patients about these vaccines in each study. Comparisons between studies are complicated by differences in HCP roles and responsibilities regarding recommendation/administration of vaccinations during pregnancy, differing national guidelines and study methodologies (e.g. capturing whether vaccines were mentioned/discussed versus recommended) and some very low response rates, suggesting that HCP samples may be non-representative with respect to vaccine recommendation behaviors.

**Table 1:** Self-reported behavior of HCPs in recommending influenza and pertussis vaccinations to pregnant women, or discussing with or informing pregnant women about these vaccinations.

Setting	HCP *n* and group	Vaccine	% HCPs who recommended vaccination to pregnant women, or who discussed with or informed pregnant women about vaccinations	Reference
**Studies that reported on HCP recommendations to pregnant women**				
Israel Multi-site (6 hospitals in Northern and Central Israel)	**150 HCPs:** 54% gynecologists 25% family practitioners 21% Master of Public Health students who work in medical system	Pertussis	68% implemented recommendation	(32)
		Influenza	70% implemented recommendation	
**US** Multi-site (Texas, New York, Illinois, Pennsylvania)	**76 HCPs:** Included ob-gyn, nurse practitioners, physician assistants, nurse midwives	Influenza	All recommended vaccine (90.7% in any trimester; 9.3% after first trimester)	(15)
**US** Pennsylvania	**24 HCPs:** All obstetric care providers	Pertussis	All said that they recommended	(25)
**France** Paris	**208 HCPs:** All midwives	Influenza	81% ever informed their patients that a vaccine was available; 17% systematically recommended that their patients were vaccinated.	(33)
**Georgia** Multi-site (8 cities: Tbilisi, Rustavi, Batumi, Caspi, Kutaisi, Tskaltubo, Gori and Kobuleti)	**278 HCPs:** All ob-gyn	Influenza	43% recommended influenza vaccination during pregnancy; 18% reported vaccinating any pregnant patients during last influenza season	(34)
**Spain** Catalonia region	**194 HCPs:** 70% midwives 30% ob-gyn	Influenza	40.8% of ob-gyn and 44.1% of midwives recommended during first trimester 85.7% of ob-gyn and 84.8% of midwives recommended during second/third trimester	(35)
		Pertussis	95.9% of ob-gyn and 97.9% of midwives recommended	
**Belgium** Flanders region	**261 HCPs:** 61% GPs 29% midwives 10% gynecologists	Influenza	72% recommend always, 11.1% sometimes	(19)
		Pertussis	75.1% recommend always, 6.1% sometimes	
**France** Loire-Atlantique	**694 HCPs:** 57% physicians, family or general medicine; 10% physicians, ob-gyn; 22% midwives; 8% midwifery students 4% unknown	Pertussis	93% indicated would follow recommendation for anti-pertussis vaccination of pregnant women if this was introduced in France	(18)
**Studies that reported on HCPs discussing with or informing pregnant women about vaccinations**				
**Ireland** County Cavan	**50 HCPs:** 70% midwives 30% hospital doctors	Pertussis and influenza	52% ever discussed both vaccinations with antenatal patients during consultations	(27)
**Germany** National	**867 HCPs:** Gynecologists in private practice	Pertussis	82% informed pregnant patients about vaccine (of these, 18.6% on patient request only)	(36)
		Influenza	98.5% informed pregnant patients about vaccine (8.6% of these on patient request only)	

Overall, studies showed that HCPs’ awareness of guidelines did not consistently translate into recommendations to pregnant women. In a study in Israel, over a quarter of 150 HCP respondents indicated that they did not recommend influenza and pertussis vaccines to pregnant women despite awareness of their recommendation in guidelines (32) while among 208 midwives in France, 91% were aware that vaccination against influenza was recommended during pregnancy but only 17% recommended this systematically (33). Among 50 HCPs in Ireland (70% midwives), 48% never discussed these vaccines with pregnant patients despite almost all being aware that guidelines existed (27). Conversely, a study in Germany found that although lack of an official recommendation about pertussis vaccination in pregnant women was the main barrier to providing vaccination (cited by 40% of HCPs), 59% reported administering vaccines anyway (36).

Several studies found that midwives were less likely to discuss vaccinations with pregnant women and recommend these than other HCPs, as were less experienced HCPs; e.g. in a French study, 42% of midwives recommended maternal influenza vaccination versus 63% of other HCPs (18), a study in Belgium found that while 78% of gynecologists and GPs recommended both influenza and pertussis vaccines, this was true for only 24% of midwives (19), while another study of midwives in France found that 50% of those with at least ten years of experience often or always suggested influenza vaccine compared with only 29% of those less experienced (33).

## Pregnant Women: Knowledge and Information Provision

Recently published studies have identified some important knowledge gaps among pregnant women regarding vaccines in pregnancy (including availability). In a multi-center Italian study, 44% and 49% of women unvaccinated for influenza and pertussis respectively were unaware that vaccination in pregnancy would provide protection for their baby from the infection in early life, and receipt of vaccination was associated with such knowledge (21), whilst single center surveys in Rome, Italy and Riyadh, Saudi Arabia found that 35% and 46% of pregnant women respectively were unaware of the elevated risk of complications associated with influenza in pregnancy (30, 37). However, this lack of understanding should be considered as a marker of the absence of advice and recommendation from HCPs (as for 82% of women in the multi-center Italian study and 99% and 97% respectively in the Rome and Riyadh surveys) rather than as an important barrier to vaccine acceptance per se.

Several recent studies have examined information provision on vaccination in pregnancy, including how this should be disseminated. In the Rome survey above, only 6% of pregnant women correctly identified the current national recommendation for influenza vaccination, despite this survey being conducted during a vaccination campaign (30). A small Canadian mixed-methods study found that around 40% of women who did not receive the influenza vaccine, including some who had intended to be vaccinated, reported not having enough information to make a decision (28). Similarly, in a large survey in Taiwan, 55% of recently delivered women who declined Tdap vaccination said that they had received insufficient information to make an informed decision and 77% said that they did not trust the information they had been given (22), whilst an Irish study found that 59% of unvaccinated women stated that inadequate information was a reason for their lack of pertussis vaccine uptake (27). In contrast, only 16% and 7% of pregnant women who intended not to receive pertussis or influenza vaccination respectively in the UK cited insufficient information as a reason (24).

Regarding information provision, a generally negative response to leaflets was found in a qualitative study in Northern Ireland, with preference for face-to-face discussion with a HCP, although most felt that insufficient time was given by HCPs for such discussions and some reported that their HCP was unable to address all their questions (38). Studies reported that pregnant women obtained information on vaccines from the media, family and friends, plus HCPs. The latter were the most common source in both a large French study of pertussis vaccination (18) and a study in New Zealand addressing influenza vaccine uptake (14). The importance of family, friends and the media as information sources among unvaccinated women varied by setting: in the New Zealand study, 20-25% cited these as having influenced their decision (14), whilst fewer than 5% of pregnant women intending not to be vaccinated in a UK study cited concerns about information in the media or the influence of family and friends as a reason (24).

Recent studies have shown sometimes significant knowledge gaps among HCPs regarding maternal immunization (18, 27, 33). Confidence to advise pregnant women also differed by profession and experience; e.g. in a UK study, only 59% of HCPs overall were extremely/moderately confident to advise pregnant women on influenza vaccine and 57% for pertussis, with midwives less confident than obstetricians (55% vs 68%) (24). In a French study, 37% of midwives self-reported limited knowledge of influenza vaccination, of whom only 13% proposed the vaccine to patients, as compared with 90% of the 9% who self-reported high knowledge (33). Only 43% of almost 300 obstetricians/gynecologists in a study in Georgia recommended influenza vaccination to patients, with 75% stating that there was insufficient evidence to support vaccination, but 93% were receptive to receiving additional education (34). Over 90% of 194 maternal care providers in Spain (mostly midwives) agreed that vaccination training for HCPs could be a strategy to improve uptake of vaccines (along with official recommendations) (35). The need for effective communication is underscored by one US study in which all HCPs reported recommending vaccination but only 85% of women reported receiving this (15); time needed to effectively counsel women about vaccination was perceived as a barrier to recommendation by HCPs in some studies (35, 36).

## Safety Concerns as a Barrier to Uptake

A consistent barrier to vaccine uptake across studies was the fear of potential harm to woman or baby. Among recent studies where reasons for declining vaccination in pregnancy were examined, the proportion of women citing concerns about safety as influencing this decision varied substantially (**Table 2**). Maternal perception of the frequency of vaccine complications was associated with uptake in a French study (17): uptake was 55% among women who thought frequency of fetal/infant complications was very low compared with 35% in those who thought these were very common, but lowest uptake (21%) was in women who thought there was a medium rate of complications. Such findings are an important reminder that some pregnant women accept vaccination despite safety concerns. This was also highlighted by a mixed methods study in Canada, with the authors noting that for most women “the unknown risks from the vaccine did not outweigh the benefits of vaccination” (28); focus groups also identified concerns regarding potential delayed discovery of vaccine-related adverse effects, consistent with another qualitative study in Northern Ireland where some unvaccinated women were worried about long-term adverse effects (38). The latter study also found that maternal vaccination was thought by some to be inconsistent with warnings around using medications whilst pregnant. An Australian survey found that fewer migrant women (comprising 69% of the sample) believed that Tdap is safe during pregnancy than Australian-born women (53% versus 65%, *p*=0.01) and that, overall, maternal belief that the vaccine was safe for the baby was the key factor associated with uptake (23).

**Table 2:** Prevalence of safety concerns as a reason given by women for not taking up influenza and/or pertussis vaccination in pregnancy.

Setting	Number of unvaccinated women responding to survey	Vaccine	% unvaccinated women reporting safety concerns as a reason for declining vaccination			Reference
			For woman	For fetus/infant	Non-specified	
**Australia** Melbourne	95	Influenza		10%		(23)
	46	Pertussis		7%	
**France** Lille	1320	Influenza	13%	24%		(17)
**Greece** Athens	164	Influenza or Pertussis	2%	8%		(20)
**Italy** Multi-center (Milan, Rome, Jesi)	682	Influenza or Pertussis		17%		(21)
**New Zealand** Wellington	16	Influenza	38%	31%		(14)
**Spain** Valencian Community	262	Influenza			21%	(16)
**Taiwan** Multi-site (8 hospitals)	473	Pertussis		44%		(22)
**UK** Multi-site (Southampton, Bristol, Oxford, London)	68	Influenza*	29%	31%		(24)
	24	Pertussis*	28%	52%	
**USA** Pennsylvania	91	Influenza*			17%	(25)
	43	Pertussis*			12%

*Women indicating intent not to vaccinate.

Safety concerns among HCPs are also barriers to vaccine recommendation. In a study of HCPs in Israel, around a third reported that Tdap and influenza vaccines were unsafe in pregnancy or controversial (32), while in a US study a similar proportion (32%) reported being concerned or very concerned about the safety of influenza vaccine in the first trimester (15). Among around 200 midwives in France, only 73% agreed that influenza vaccine was safe in pregnancy; 39% had been vaccinated themselves and this group were more likely to recommend vaccination to patients (33). Among HCPs giving reasons for not recommending vaccines in pregnancy in a study in Spain, concerns relating to adverse events were more common among midwives than obstetricians/gynecologists (30.8% vs 10% respectively) (35). Of note, in one US study 71% of obstetric care providers were concerned about the safety of influenza vaccination in the first trimester and 46% about the safety of Tdap, but all still recommended vaccination, indicating that as for women, concerns about safety do not necessarily preclude vaccination recommendations.

## Perception of Risk of Infection, and Severity, in Pregnancy and Infancy

Perception of susceptibility and severity are constructs that influence health behaviors according to the Health Belief Model. Lefebvre and colleagues in a French study reported that women who perceived risk of acquisition of pertussis to be non-existent or low were significantly less likely to accept vaccination than those who perceived risk to be high (adjusted OR 0.44 [0.31,0.62]) (18). Similarly, in the Taiwanese study discussed above, 18% of women declining pertussis vaccination reported that the main factor in their decision was their belief that pertussis is not a severe disease in newborn infants; conversely, multivariable analysis showed that rating pertussis among young infants as highly severe was significantly associated with acceptance of the vaccine (22). A Canadian study also found that women’s opinions on vulnerability to influenza and its severity were central factors regarding uptake, with most women in this qualitative study not perceiving themselves or their infants to be at high risk of infection. Of note, there was sub-group of women who noted their increased vulnerability (e.g. due to occupational exposure or because of conditions such as asthma) and this group had high vaccine uptake (28).

## Other Factors Influencing Acceptance

Prior vaccination experience was an important factor influencing uptake in several studies, both with vaccine-experienced women being more likely to take up influenza vaccination (17, 21) and with history of no previous vaccination and/or past refusal being associated with non-acceptance of vaccines in pregnancy (16, 18). With respect to maternal socio-demographics, higher maternal education level was associated with pertussis but not with influenza vaccine uptake in two studies (21, 23), while a Belgian study found education level to be associated with coverage of both vaccines (19). Another study found that parity was associated with uptake, with women with two or more previous deliveries less likely to receive influenza vaccine than women with fewer (17), possibly reflecting access challenge relating to childcare responsibilities.

## COVID-19 Considerations

Vaccine development is essential to the COVID-19 response, with rapid progress of Phase III clinical trials (all excluding pregnant women), licensing and roll-out (39, 40). The pandemic may modify perceptions and/or health seeking behaviours regarding vaccination for respiratory infections, as shown by a study examining online interest in COVID-19 and vaccinations worldwide through Google Trends, which found an upsurge in interest in influenza and pneumococcal vaccines concurrent with the first pandemic wave (41). An important impact of COVID-19 on vaccination to date has been the world-wide disruption to routine immunisations for reasons including reduced access to services during lockdowns, HCP capacity issues, reluctance to attend health services for vaccinations (e.g., due to fears about exposure to SARS-CoV-2, or due to confusing messaging around “protecting” health services) (42–44). Results from an international survey of clinicians in April 2020 showed that 50% of respondents had problems regarding maternal immunisation delivery (43). More research is needed to understand the collateral damage inflicted by the pandemic on maternal immunisation, as the impact on vaccination rates remains unknown. There is also the question of COVID-19 vaccination in pregnancy, currently a focus of the Pregnancy Research Ethics for Vaccines, Epidemics and New Technologies (PREVENT) group (45). In the absence of trial data among pregnant women, guidance from governments and professional bodies is highly variable with respect to pregnancy and vaccination, and subject to change; many currently recommend an individual risk-benefit approach which is challenging given the evidence gap (46). The recent announcement of a Phase 2/3 study to assess the safety, tolerability, and immunogenicity of the Pfizer-BioNTech COVID-19 vaccine (BNT162b2) in preventing COVID-19 in healthy pregnant women is therefore very welcome (ClinicalTrials.gov Identifier: NCT04754594).

## Discussion

Understanding reasons for vaccine hesitancy and/or low coverage in pregnancy (which may be related to the individual woman, to the vaccinator, to policies or to structural factors) is a pre-requisite for addressing them. The recent studies examined here have provided useful information for policymaking in vaccine delivery. However, study limitations should be considered, including a high proportion of single site studies, use of convenience samples and general reliance on self-reported vaccination status. The findings also underscore the importance of context, with highly variable uptake rates reported ranging from 0% to 78%. This limits comparisons between studies and precludes summary estimates of vaccine uptake or of HCP provider behavior such as vaccine recommendation.

Despite maternal vaccines for influenza and pertussis being safe and effective, safety concerns among women and some HCPs are well-established barriers to uptake/coverage (13). Recent studies have continued to examine perceptions and beliefs of pregnant women regarding vaccine safety for themselves and/or their baby. Between 7% and 52% of unvaccinated women gave safety concerns as a reason for decline in reviewed studies, but some did not investigate the association between presence of safety concerns and actual uptake. Qualitative studies tended to provide richer data on the precise nature of women’s concerns whilst overall findings underscored that maternal worries about safety are not necessarily incompatible with acceptance of a vaccine in pregnancy. A greater understanding of what facilitates HCP recommendation of vaccination in pregnancy and what prevents them from doing so in different settings/contexts is needed. More research on specific factors shaping maternal confidence in vaccines, to incorporate the potential influence of COVID-19, is also required.

## Author Contributions

XQ, HB and CT developed the search strategy, XQ conducted the literature search and did the initial screen of articles. XQ, HB and CT wrote the manuscript together. All authors contributed to the article and approved the submitted version.

## Funding

This work is partly funded by the NIHR GOSH BRC. The views expressed are those of the author(s) and not necessarily those of the NHS, the NIHR or the Department of Health.

## Conflict of Interest

The authors declare that the research was conducted in the absence of any commercial or financial relationships that could be construed as a potential conflict of interest.
